# Supplementary material for: Single-mRNA imaging and modeling reveal coupled translation initiation and elongation rates
Source: eLife. 2026 Apr 17;14:RP107160. doi: 10.7554/eLife.107160 (PMC13090027; doi:10.7554/eLife.107160)
Supplement: Supplementary file 1. [file elife-107160-supp1.docx]

1. COL1A1_sub: A 130 T 225 C 357 G 344 | GC%: 66.38% | Length: 1056

GCTACTGGTTTCCCTGGTGCTGCTGGCCGAGTCGGTCCTCCTGGCCCCTCTGGAAATGCTGGACCCCCTGGCCCTCCTGGTCCTGCTGGCAAAGAAGGCGGCAAAGGTCCCCGTGGTGAGACTGGCCCTGCTGGACGTCCTGGTGAAGTTGGTCCCCCTGGTCCCCCTGGCCCTGCTGGCGAGAAAGGATCCCCTGGTGCTGATGGTCCTGCTGGTGCTCCTGGTACTCCCGGGCCTCAAGGTATTGCTGGACAGCGTGGTGTGGTCGGCCTGCCTGGTCAGAGAGGAGAGAGAGGCTTCCCTGGTCTTCCTGGCCCCTCTGGTGAACCTGGCAAACAAGGTCCCTCTGGAGCAAGTGGTGAACGTGGTCCCCCTGGTCCCATGGGCCCCCCTGGATTGGCTGGACCCCCTGGTGAATCTGGACGTGAGGGGGCTCCTGGTGCCGAAGGTTCCCCTGGACGAGACGGTTCTCCTGGCGCCAAGGGTGACCGTGGTGAGACCGGCCCCGCTGGACCCCCTGGTGCTCCTGGTGCTCCTGGTGCCCCTGGCCCCGTTGGCCCTGCTGGCAAGAGTGGTGATCGTGGTGAGACTGGTCCTGCTGGTCCCGCCGGTCCTGTCGGCCCTGTTGGCGCCCGTGGCCCCGCCGGACCCCAAGGCCCCCGTGGTGACAAGGGTGAGACAGGCGAACAGGGCGACAGAGGCATAAAGGGTCACCGTGGCTTCTCTGGCCTCCAGGGTCCCCCTGGCCCTCCTGGCTCTCCTGGTGAACAAGGTCCCTCTGGAGCCTCTGGTCCTGCTGGTCCCCGAGGTCCCCCTGGCTCTGCTGGTGCTCCTGGCAAAGATGGACTCAACGGTCTCCCTGGCCCCATTGGGCCCCCTGGTCCTCGCGGTCGCACTGGTGATGCTGGTCCTGTTGGTCCCCCCGGCCCTCCTGGACCTCCTGGTCCCCCTGGTCCTCCCAGCGCTGGTTTCGACTTCAGCTTCCTGCCCCAGCCACCTCAAGAGAAGGCTCACGATGGTGGCCGCTACTACCGGGCTGATGATGCCAATGTGGTT

2. COL1A1_sub_PtoA: A 132 T 234 C 264 G 426 | GC%: 65.34% | Length: 1056

GCTACTGGTTTCCCTGGTGCTGCTGGCCGAGTCGGTGCTGCTGGCGCTTCTGGAAATGCTGGAGCTGCTGGCGCTGCCGGTGCTGCTGGCAAAGAAGGCGGCAAAGGTGCCCGTGGTGAGACTGGCGCTGCTGGACGTGCTGGTGAAGTTGGTGCCGCCGGTGCCGCTGGCGCTGCTGGCGAGAAAGGATCCGCTGGTGCTGATGGTGCTGCTGGTGCTGCTGGTACTGCTGGGGCCCAAGGTATTGCTGGACAGCGTGGTGTGGTCGGCCTGGCTGGTCAGAGAGGAGAGAGAGGCTTCGCTGGTCTTGCTGGCGCTTCTGGTGAAGCTGGCAAACAAGGTGCTTCTGGAGCAAGTGGTGAACGTGGTGCCGCCGGTGCTATGGGCGCTGCCGGATTGGCTGGAGCTGCTGGTGAATCTGGACGTGAGGGGGCTGCCGGTGCCGAAGGTTCCGCCGGACGAGACGGTTCTGCTGGCGCCAAGGGTGACCGTGGTGAGACCGGCGCTGCTGGAGCCGCTGGTGCTGCAGGTGCTGCTGGTGCCGCTGGCGCTGTTGGCGCTGCTGGCAAGAGTGGTGATCGTGGTGAGACTGGTGCCGCTGGTGCTGCCGGTGCTGTCGGCGCTGTTGGCGCCCGTGGCGCTGCCGGAGCTCAAGGCGCTCGTGGTGACAAGGGTGAGACAGGCGAACAGGGCGACAGAGGCATAAAGGGTCACCGTGGCTTCTCTGGCCTCCAGGGTGCTGCTGGCGCTGCTGGCTCTGCCGGTGAACAAGGTGCTTCTGGAGCCTCTGGTGCCGCTGGTGCTCGAGGTGCTGCCGGCTCTGCTGGTGCTGCTGGCAAAGATGGACTCAACGGTCTCGCTGGCGCTATTGGGGCAGCTGGTGCTCGCGGTCGCACTGGTGATGCTGGTGCTGTTGGTGCTGCCGGCGCCGCTGGAGCTGCCGGTGCTGCTGGTGCTGCTAGCGCTGGTTTCGACTTCAGCTTCCTGGCTCAGGCAGCTCAAGAGAAGGCTCACGATGGTGGCCGCTACTACCGGGCTGATGATGCCAATGTGGTT
